# Community perceptions of malaria and malaria treatment behaviour in a rural district of Ghana: implications for artemisinin combination therapy

**DOI:** 10.1186/1471-2458-10-409

**Published:** 2010-07-12

**Authors:** Kwaku P Asante, Livesy Abokyi, Charles Zandoh, Ruth Owusu, Elizabeth Awini, Abubakari Sulemana, Seeba Amenga-Etego, Robert Adda, Owusu Boahen, Sylvester Segbaya, Emmanuel Mahama, Constance Bart-Plange, Daniel Chandramohan, Seth Owusu-Agyei

**Affiliations:** 1 Kintampo Health Research Centre, Ghana Health Service, P. O. Box 200, Kintampo, Ghana; 2 Dodowa Health Research Centre, Ghana Health Service, P.O. Box 1 Dodowa, Dangme West District, Accra Region, Ghana; 3 National Malaria Control Programme, Ghana Health Service, P. O. Box KB 493, Korle-Bu, Accra, Ghana; 4 Infectious and Tropical Diseases Department, London School of Hygiene & Tropical Medicine, Keppel, WC1E 7HT St London, UK

## Abstract

**Background:**

Artesunate-amodiaquine (AS-AQ) was introduced in Ghana as the first line drug for treatment of uncomplicated malaria in 2004. We report the perceptions of malaria and malaria treatment behaviour, the community awareness of and perceptions about AS-AQ two years after the introduction of this ACT treatment for malaria.

**Methods:**

Two surveys were conducted; a cross-sectional survey of 729 randomly selected household heads (urban-362, rural-367) and 282 women with children < 5 years (urban-121, rural-161) was conducted in 2006. A district wide survey was conducted in 2007 to assess awareness of AS-AQ. These were complemented with twenty-eight focus group discussions (FGDs) and 16 key informant interviews (KII) among community members and major stakeholders in the health care delivery services. All nine (9) health facilities and five (5) purposively selected drug stores were audited in order to identify commonly used anti-malarials in the study area at the time of the survey.

**Results:**

Majority of respondents ( > 75%) in the sampled survey mentioned mosquito bites as the cause of malaria. Other causes mentioned include environmental factors (e.g. dirty surroundings) and standing in the sun. Close to 60% of the household heads and 40% of the care-givers interviewed did not know about AS-AQ. The community respondents who knew about and had ever taken AS-AQ perceived it to be a good drug; although they mentioned they had experienced some side effects including headaches and body weakness. Co-blistered AS-AQ was available in all the government health facilities in the study area. Different formulations of ACTs were however found in urban chemical shops but not in rural chemical stores where monotherapy antimalarials were predominant.

**Conclusion:**

The knowledge of fever as a symptom of malaria is high among the study population. The awareness of AS-AQ therapy and its side-effect was low in the study area. Community education and sensitization, targeting all categories of the population, has to be intensified to ensure an efficient implementation process.

## Background

It has been estimated that about 3000 malaria deaths occur among African children each day [1], with about 0.5 billion clinical malaria cases and 2-3 million severe malaria episodes occurring annually [2]. The children who do not die from the severe form of malaria may suffer brain damage or experience cognitive and learning deficits [3]. The World Bank ranked malaria as a leading cause of most disability-adjusted life years in Africa with an estimated 35 million future life-years lost from disability and premature deaths [4]. The loss of growth in countries with endemic malaria is estimated at about 12 billion US Dollars annually [5]. This has contributed to the cycle of poverty in sub-Saharan Africa [6].

Poor perceptions about malaria and poor malaria drug treatment practices have contributed to widespread resistance of *Plasmodium falciparum *malaria to commonly used monotherapy such as chloroquine and sulfadoxine-pyrimethamine leading to challenges in the control of malaria [7]. Artemisinin-based combination therapy (ACT) has been demonstrated to remarkably improve treatment efficacy [8] and are currently recommended by the World Health Organization to overcome the problem of drug resistance and for effective control of malaria [9].

Current efforts to eliminate malaria greatly depend on the perceptions of anti-malarial interventions. The perceptions of malaria and community treatment seeking behaviour have been widely investigated in relation to monotherapies, mainly chloroquine and sulphadoxine-pyrimethamine in Ghana and other African countries. Understanding the perceptions of malaria, treatment behaviour, awareness of and prompt access to the new and effective ACT drugs are deemed important steps to malaria control and elimination.

In Ghana, public education on causes, prevention, and actions for treatment of malaria has been ongoing for several years. Such educational campaigns are even more crucial now to maintain the effectiveness of ACTs. In 2004, Ghana changed its firstline malaria treatment policy from chloroquine to the use of artesunate-amodiaquine (AS-AQ) combination. The policy change process involved active education campaign through media advertisements, community durbars and workshops among health-workers.

We conducted two surveys in the Kintampo North and South Districts of Ghana; the first was between March. 2006 and July 2006 and a repeat in June 2007 in order to determine the perceptions of malaria and malaria treatment behaviours. We also assessed the awareness of and perceptions about AS-AQ in the communities two years after it was introduced as the first line drug for treatment of malaria.

## Methods

### Study site

The study was carried out in the Kintampo North and South Districts of Ghana. These two districts cover in total, an area of 7162 km^2 ^with a resident population of approximately 130,000. The study area is located within the forest-savannah transitional ecological zone in central Ghana where community members are predominantly subsistent farmers. *Plasmodium falciparum *malaria predominates in this area. Community surveys showed the prevalence of malaria parasitemia to be about 50% among children less than 10 years of age (symptomatic/asymptomatic) and malaria transmission is perennial, with entomological inoculation rate of 269 infective bites per person per year [10]. Mean monthly temperatures range between 18°C to 38°C and rainfall averages 1250 mm per annum, making conditions optimal for vector abundance. *Plasmodium falciparum *resistance to chloroquine was greater than 40.0%, similar to that recorded in other parts of the country [11]. Seven (7) clinics and two (2) hospitals are located in the two districts. Malaria microscopy is available for malaria diagnosis in the hospitals. Malaria Rapid Diagnostic Test (RDT) kits are supposed to be used in peripheral clinics. Malaria diagnosis at the chemical shops is mainly presumptive. Antimalarials for uncomplicated malaria are registered to be sold over the counter in the study area and other parts of Ghana.

### Quantitative survey

Two surveys were conducted; the first survey was between March 2006 and July 2006 among seven hundred and twenty-nine (729) randomly selected households within the study area, using the urban/rural strata. The household database of the Kintampo Health and Demographic Surveillance Systems (KHDSS) was used as a sampling frame. A maximum of three persons made up of the household head, a woman with a child less than five years and a household member who had taken AS-AQ prior to the survey were interviewed in each household. Trained field workers interviewed respondents about their perceptions of malaria, malaria treatment behaviours, and the awareness of AS-AQ therapy as first line antimalarial using structured questionnaires. Malaria treatment behaviours were assessed with reference to the last episode of malaria experienced by the respondent in the last two months. Respondents were made to identify the types/brands of antimalarials displayed from a collection of antimalarials available in the study area; these included AS-AQ brands that they were supposed to be aware of or had taken prior to this survey.

The second survey was a district-wide household survey conducted from January to June 2007 involving the entire households in the Kintampo Health and Demographic Surveillance System (KHDSS) in order to further assess the awareness of AS-AQ. Respondents to questions during this survey were household heads or their representatives. The KHDSS is a system that conducts six (6)-monthly censuses in the entire population of Kintampo North and South districts in the central part of Ghana. It generates data from the entire population that informs public health policy in Ghana. In both surveys, awareness of AS-AQ was defined as ability to mention the name of AS-AQ or identify AS-AQ as a replacement of chloroquine for the treatment of malaria.

### Qualitative methods

Twenty-eight focus group discussions (FGDs) were conducted among community members. The FGDs assessed local names for the syndrome of signs and symptoms that describe malaria and its treatment practices. Responses were used to develop the quantitative survey tools. FGD participants from rural and urban areas of the study were randomly selected using a list from KHDSS database of community resident members. In each area, selected participants were categorised based on sex, age groups, and women with children less than five years of age. Each FGD session involving between 8 to 12 participants was moderated by a study team member as well as recorded for transcription and analysis.

Nineteen (19) key informant interviews (KII) were also conducted among stakeholders in healthcare delivery including; a District Director of Health Services, nine (9) primary doctor/medical assistants located at all the nine (9) health facilities in the study area. Additionally, four (4) traditional healers and five (5) out of seventeen (17) drug store operators were selected in the study area based on their accessibility and availability to community members in the study area. By the fifth interview with a drug store operator, there were no new emerging themes being obtained as determined by continuous analysis of already conducted interviews. Drug stores have similar characteristics such as personnel who manage them and their scope of work. The shops are managed by community members without qualified pharmacist. They are the lowest level of pharmaceutical care providers who are allowed to supply only over-the-counter medicines by retail. The shops are licensed and regulated by the Ghana Pharmacy Council which ensures their adherence to standards of community based pharmaceutical care throughout Ghana including the study area. The interviews explored their knowledge of and perceptions about the anti-malaria policy change and methods used in identifying and reporting the side-effects of AS-AQ.

### Health facility survey

A survey of all nine (9) health facilities and five (5) purposively selected drug stores were carried out to identify commonly used anti-malarial drugs in the study area. The selection was based on their accessibility and availability to community members in the study area. Samples of these anti-malarial drugs were displayed during the group discussions and surveys.

### Data entry, cleaning and analyses

Qualitative data from FGDs and IDIs were recorded using a recorder, transcribed verbatim, checked for completeness and accuracy. Answers to qualitative questions were grouped and categorised using QRS NVIVO version 7 qualitative software. Responses were analysed to identify themes that addressed the study's objectives and emerging themes. Quotes that best described the various themes were included to support quantitative findings. Quantitative data was checked for completeness and consistency and all queries resolved after double data entry. Clean data were analysed using Stata 9.0 (TX, U.S.A). Demographic data such as educational status and sex were summarised into percentages; ages were put into age-groups and summarised as percentages.

### Ethical issues

Ethical approval to conduct the study was granted by the Ghana Health Service Ethics Review Committee and the Kintampo Health Research Centre Ethics Committee. Community entry involved explaining the study to key community opinion leaders followed by community durbars/meetings. At these meetings, the study's aims, objectives, risk and benefits were explained to all participants and a signed/thumb printed written consent was obtained from each respondent and participants of the FGDs. All data collected were kept in locked cabinets to ensure confidentiality.

## Results

There were 729 household heads, 81 household members and 282 care-givers who were respondents for the quantitative survey (Table 1). All care-giver respondents in rural and urban areas were females; majority of them had had no education; this was relatively higher in the rural areas compared with the urban areas. Respondents in the focus group discussions had similar social characteristics as respondents in the quantitative survey as they were invited from the same communities.

**Table 1:** Characteristics of respondents in 2006 household survey

	Household Heads		Household members		Care-giver	
	Urban	Rural	Urban	Rural	Urban	Rural
	N = 362	N = 367	N = 38	N = 43	N = 121	N = 161
**Sex (%)**						
% Female	45.0	29.2	76.3	72.1	100.0	100.0

**Age groups (%)**						
14 - 18	0.8	0.0	13.2	0.0	0.0	0.0
19 - 24	3.6	0.5	13.2	4.6	18.2	22.4
25 - 34	16.6	13.4	15.7	25.6	45.5	41.0
35 - 49	40.1	40.3	34.2	44.2	32.2	35.4
50 - 59	16.5	25.4	13.2	16.3	4.1	1.2
60 +	22.4	20.4	10.5	9.3	0.0	0.0

**Educational characteristics (%)**						
None	43.9	54.0	21.1	41.9	43.8	50.9
Primary	9.1	13.9	18.4	14.0	19.8	16.2
Middle School, JHS	32.9	26.7	47.3	39.5	28.9	32.3
Technical/Commercial/SHS	9.1	4.1	5.3	2.3	6.6	0.6
Post Sec./college/training	3.9	1.3	7.9	2.3	0.0	0.0
University	1.1	0.0	0.0	0.0	0.9	0.0

### Perception of Malaria and Its Causes

Malaria was identified as the most disturbing disease affecting both children and adults in the study area. The local terms associated with malaria varied based on ethnic diversity; among the majority of Akan speaking communities, the main terms used were "*ebunu"*, "*etiridii" *and "*ahobene"*. Others described malaria as *ntontom yadie *i.e. mosquito disease. The majority of respondents mentioned mosquito bites as one of the factors leading to malaria disease (Table 2). A high proportion of respondents mentioned standing or walking in the sun (range 21.1-47.1%) or eating contaminated food (range 23.1-34.2%) as factors leading to malaria. Dirty environments and weedy surroundings were mentioned less frequently by all respondents (Table 2).

**Table 2:** Factors spontaneously mentioned by respondents as the cause(s) of malaria from 2006 survey*.

	Household Head		Household Member		Care-giver	
	Urban	Rural	Urban	Rural	Urban	Rural
	N = 361	N = 365	N = 38	N = 43	N = 121	N = 161
	**%, (n, 95% CI)**	**%, (n, 95% CI)**	**%, (n, 95% CI)**	**%, (n, 95% CI)**	**%, (n, 95% CI)**	**%, (n, 95% CI)**

Mosquito bites	74.8 (270, 70.0 - 79.2)	78.9 (288, 74.4 - 83.0)	89.5 (34, 75.2 - 97.1)	76.7 (33, 61.4 - 88.2)	84.3 (102, 76.6 - 90.3)	76.4 (123, 69.1 - 82.7)
Weedy Surroundings/Stagnant water	32.1 (116, 27.3 - 37.2)	39.2 (34.1 - 44.3)	36.8 (14, 21.8 - 54.0)	32.6 (14, 19.0 - 48.5)	24.8 (30, 17.4 - 33.5)	24.2 (39, 17.8 - 31.6)
Dirty Surroundings	38.0 (137, 32.9 - 43.2)	46.3 (169, 41.0 - 51.6)	34.2 (13, 19.6 - 51.4)	55.8 (24,39.9 -70.9)	40.5 (49, 31.7 - 49.8)	32.3 (52, 25.2 - 40.1)
Eating contaminated food	23.3 (84, 19.0 - 28.0)	29.3 (107, 24.7 - 34.3)	23.7 (9, 11.4 - 40.2)	25.6 (11, 13.5 - 41.2)	23.1 (28, 16.0 - 31.7)	34.2 (55, 26.9 - 42.0)
Standing/walking in the sun	41.0 (148, 35.9 - 46.2)	47.1 (172, 41.9 - 52.4)	21.1 (8, 9.6 - 37.3)	34.9 (15, 21.0 - 50.9)	33.1 (40, 24.8 - 42.2)	36.0 (28.6 - 43.9)
Sweet foods	2.2 (8, 0.9 - 4.3)	3.6 (13, 1.9 - 6.0)	2.6 (1, 0.1 - 13.8)	7.0 (3, 1.5 - 19.1)	2.5 (3, 0.5 - 7.1)	1.2 (2, 0.2 - 4.4)

*multiple answers were allowed.

### Community knowledge about the signs and symptoms of malaria

A comprehensive list of signs and symptoms of malaria was provided by the study population (Table 3). Respondents in all the FGDs conducted mentioned hot body (especially at night), headache, restlessness, loss of appetite, bitterness in the mouth, body weakness, body and joint pains, dizziness, tired feeling, cold, chills and a feeling that makes you fond of sitting in the sun as signs and symptoms associated with malaria Yellow eyes; dark or yellow urine, and yellow vomitus were associated with severity of malaria.

**Table 3:** Knowledge of signs and symptoms of malaria among respondents from 2006 survey.

	Household head		Household member		Caregiver	
	Urban	Rural	Urban	Rural	Urban	Rural
	N = 361	N = 365	N = 38	N = 43	N = 121	N = 161
**Symptom**	**%, (n, 95% CI)**	**%, (n, 95% CI)**	**%, (n, 95% CI)**	**%, (n, 95% CI)**	**%, (n, 95% CI)**	**%, (n, 95% CI)**

Bodily Weakness	59.0 (213, 53.7 - 64.1)	62.5 (228, 57.3 - 67.5)	68.4 (26, 51.3 - 82.5)	62.8 (27, 46.7 - 77.0)	34.7 (42, 26.3 - 43.9)	31.1 (50, 24.0 - 38.8)
Hot body	46.8 (169, 41.6 - 52.1)	36.4 (133, 31.5 - 41.6)	50.0 (19, 33.3 - 66.6)	37.2 (16, 23.0 - 53.3)	82.6 (100, 74.7 - 88.9)	81.4 (131, 74.5 - 87.1)
Loss of appetite	44.3 (160, 39.1 - 49.6)	33.2 (121, 28.3 - 38.2)	50.0 (19, 33.3 - 66.6)	39.5 (17, 25.0 - 55.6)	30.6 (37, 22.5 - 39.6)	29.8 (48, 22.9 - 37.5)
Chills	40.4 (146, 35.3 - 45.7)	45.2 (165, 40.0 - 50.5)	44.7 (17, 28.6 - 61.7)	37.2 (16, 23.0 - 53.3)	21.5 (26, 14.5 - 29.9)	24.2 (39, 17.8 - 31.6)
Headache	37.1 (134, 32.1 - 42.3)	29.9 (109, 25.2 - 34.8)	36.8 (14, 21.8 - 54.0)	32.6 (14, 19.1 - 48.5)	15.7 (19, 9.7 - 23.4)	13.7 (22, 8.8 - 19.9)
Vomiting	19.7 (71, 15.7 - 24.1)	16.4 (60, 12.8 - 20.6)	15.8 (6, 6.0 - 31.2)	16.3 (7, 6.8 - 30.7)	42.2 (51, 33.2 - 51.4)	41.0 (66, 33.3 - 49.0)
Dizziness	18.8 (68, 14.9 - 23.2)	28.8 (105, 24.2 - 33.7)	13.2 (5, 4.4 - 28.1)	30.2 (13, 17.2 - 46.1)	6.6 (8, 2.9 - 12.6)	9.3 (15, 5.3 - 28.9)
Diarrhea	7.2 (26, 4.7 - 10.4)	8.5 (31, 5.8 - 11.8)	10.5 (4, 2.9 - 24.8)	9.3 (4, 2.6 - 22.1)	12.4 (15, 7.1 - 19.6)	21.7 (35, 15.6 - 28.9)
Cough	1.7 (6, 0.6 - 3.6)	1.5 (6, 0.6 - 3.5)	0.0 (0, -)	7.0 (3, 1.5 - 19.1)	3.3 (4, 0.9 - 8.2)	3.7 (6, 1.4 - 7.9)

*"Your whole body will become hot, your mouth becomes bitter and you don't have the appetite for anything" (FGD Atta Akura, female 35-49). "You will feel cold, weak, with bitterness in the mouth and when you urinate, the urine is very yellowish in colour" (FGD Sunkwa, mothers less than 30 years)*.

Most of the care-givers of children less than five years of age associated malaria with hot body (Urban -82.6% (95% CI 74.7 - 88.9); Rural -81.4% (95% CI 74.4. - 87.1) temperature when interviewed whiles adults associated body weakness with malaria (Table 3).

### Health seeking behavior among community members

Self-medication was found to be a common practice for managing most disease conditions in the study population. Community members mentioned that management of malaria is initially at home and then a follow-up to the hospital if the situation of the patient did not improve. In critical situations, community members who initially seek traditional treatment resort to treatment at the health facility. These findings were apparent from comments made in the focus group discussions:

"What happens is, you have to use some local herbs and see but when it becomes severe then you go to the hospital." (FGD mothers less than 30 years, Kokuma)

"*Most often, we usually manage the sickness at home a little. We are able to manage it by giving for instance paracetamol syrup. If we see that after some days the situation is not improving then we take the child to the hospital" (IDI mother above 30 years)*

Participants who use orthodox medicines usually resort to the use of analgesics, antipyretics and antimalarials which they usually obtain from drug vendors/peddlers or licensed chemical shops as the first treatment option. Left over drugs previously used to treat similar conditions were also used for treating a patient with another onset of malaria. Participants also use home-made herbal preparations from different plant parts. In some cases, herbal preparations peddled by traditional healers or sold in shops are used. These preparations are boiled and drunk, inhaled or bathed. The potency of some of these preparations is determined by how frequent one urinates. If one urinates a lot after taking these herbs, it is believed that the treatment is good and that the sickness is being passed out through the urine.

"I also know of "dua gene" the Ashantis call it "gyedua", you add "nsempuadua" leaves and cassava leaves. You boil them together, and then drink some and bath some. You will realize that the 'fever' will go. If I drink it, I will urinate all the 'fever' away" (FGD Jema East males 50 years and above)

There was the perception among respondents that traditional treatment worked better for them whilst others believed in orthodox treatment. In both instances, the effect of treatment was associated with one's body including their 'blood' (a description referable to the hematological system) and its compatibility with whichever drug taken.

"For me if I get malaria, I can go to the hospital ten times but it would not go, unless I use herbs before it will go."(FGD Mo line females 25-34 years)

"If the child's body is hot, go for mango or pawpaw leaves and boil it and give it to the child. So when you do that his body will cool down." (FGD 19-24 females, Kandige)

Chemical shops were found to be a common place where respondents first go for advice on what drugs to buy after describing their sick conditions to the chemical seller. In the rural areas where drug peddlers are well patronized, drugs including antimalarials, analgesics, and antibiotics are sold. These health seeking practices were supported in the quantitative survey (Table 4).

**Table 4:** First point of call when the respondents are sick from 2006 survey.

	Household heads		Household members		Care-givers	
	Urban	Rural	Urban	Rural	Urban	Rural
	N = 275	N = 280	N = 33	N = 32	N = 95	N = 135
	**%, (n, 95% CI)**	**%, (n, 95% CI)**	**%, (n, 95% CI)**	**%, (n, 95% CI)**	**%, (n, 95% CI)**	**%, (n, 95% CI)**

Chemical Shop	51.6 (142, 45.6 - 57.7)	41.8 (117, 35.9 - 47.8)	30.3 (10, 15.6 - 48.7)	40.6 (13, 23.7 - 59.4)	44.2 (42, 34.0 - 54.8)	36.3, (49, 28.2 - 45.0)
Hospital	18.2 (50, 13.8 - 23.3)	15.4 (43, 11.3 - 20.1)	24.2 (8, 11.0 - 42.2)	25.0 (8, 11.4 - 43.4)	21.1 (20, 13.3 - 30.6)	17.0 (23, 11.1 - 24.5)
Use of traditional medicine	10.9 (30, 7.4 - 15.2)	23.2 (65, 18.3 - 28.6)	9.1 (3, 1.9 - 24.3)	9.4 (3, 2.0 - 25.0)	1.0 (1, 0.0 - 5.7)	9.6 (13, 5.2 - 16.0)
Health Centre	8.0 (22, 5.1 - 11.9)	7.1 (20, 4.4 - 10.8)	24.2 (8, 11.1 - 42.2)	12.5 (4, 3.5 - 29.0)	13.7 (13, 7.5 - 22.3)	12.6 (17, 7.5 - 19.4)
Private Clinic	2.9 (8, 1.3 - 5.6)	4.3 (12, 2.2 - 7.4)	3.1 (1, 0.1 - 15.7)	0.0 (0, -)	9.5 (9, 4.4 - 17.2)	2.2 (3, 0.5 - 6.3)
Use of left over orthodox medicine at home	2.9 (8, 1.3 - 5.6)	4.3 (12, 2.2 - 7.4)	9.1 (3, 1.9 - 24.3)	3.1 (1, 0.1 - 16.2)	9.5 (9, 4.4 - 17.2)	10.4 (14, 5.8 - 16.8)
Drugs from Friend/relative	2.6 (7, 1.0 - 5.2)	0.4 (1, 0.0 - 1.9)	0.0 (0, -)	0.0 (0, -)	1.0 (1, 0.0 - 5.7)	3.0 (4, 0.8 - 7.4)
Herbalist	0.7 (2, 0.1 - 2.6)	0.7 (2, 0.1 - 2.5)	0.0 (0 - )	0.0 (0, -)	0.0 (0, -)	0.7 (1, 0.0 - 4.0)
Other	2.2 (6, 0.8 - 4.7)	2.9 (8, 1.2 - 5.5)	0.0 (0 - )	9.4 (3, 2.0 - 25)	0.0 (0, -)	8.2 (11, 4.1 - 14.1)
**Total**	**100**	**100.0**	**100.0**	**100**	**100.0**	**100.0**

The community's health seeking practices were further affirmed by a health worker in an IDI; she was of the view that community members initially report at chemical shops for drugs when they get sick and only report at the health facility when the illness gets complicated.

Respondents attached importance in seeking health care at the health facility; lack of money for such services was however considered a major barrier for early treatment at the health facility as demonstrated in the statement;

*"Normally we need the hospital drugs, but sometimes when the sickness comes, the money you need to go to the hospital becomes difficult to get so you go for the leaves" (FGD Atta Akura female 35-49 years)*.

### Awareness of AS-AQ among sampled households and all households in the study area

Awareness of AS-AQ was generally low among the sampled population in both rural and urban areas (Table 5). Among household heads, urban awareness was 33.2% (95% CI 28.2 - 38.3) compared with the rural folks 32.6% (95% CI 27.9 - 37.8); caregivers in the urban parts had awareness levels of 39.5% (95% CI 30.9 - 48.9) compared with rural folks, 46.5% (95% CI 38.7 - 54.6). Awareness of AS-AQ was much lower in both urban and rural areas when the district-wide survey was conducted among all households or their representatives in the study area. The urban awareness was 25.8% (95% CI 24.6 - 27.0) compared to rural -6.9% (95% CI 6.4 - 7.5).

**Table 5:** Proportion of respondents who were aware of AS-AQ among sampled population in 2006 and district wide survey in 2007

Sampled population in 2006	N	%	95% CI
Household head			
Urban (N = 361)	120	33.2	28.2 - 38.3
Rural (N = 365)	119	32.6	27.9 - 37.8

Care giver			
Urban (N = 121)	48	39.6	30.9 - 48.9
Rural (N = 160)	74	46.3	38.7 - 54.6

District wide population 2007			
Urban (N = 5339)	1378	25.8	24.6 - 27.0
Rural (N = 9787)	683	6.9	6.4 - 7.5

Community members who were aware of AS-AQ reported of diverse sources of their information about AS-AQ, the commonest being the local radio and/or the National Television stations. About sixty seven percent (66.7%, 95% CI 57.5 - 75.0) of household heads in the urban areas and 48.7% (95% CI 39.4 - 58.1) of household heads in the rural areas reported the radio and television as the main sources of information; similar responses were solicited from care-givers where information among the urban population was 52.6% (95% CI 35.8 - 69.0) compared with the rural folks 34.8% (95% CI 21.0 - 50.9). Other sources of information about AS-AQ among caregivers included health talk sessions during child welfare clinics, health talk at churches, and discussions with relatives or in chemical shops owners. In rural areas,17.6% (95% CI 11.3 - 25.7) of household heads and 37.2% (95% CI 23.0 - 53.3) care-givers had heard of AS-AQ during community durbars organised by health workers.

Suggestions on ways to promote awareness of AS-AQ were assessed in community FGDs. Participants in the discussions who were hearing about AS-AQ for the first time were ready to learn about it and tell others. Radio discussions, community durbars and church announcements were methods suggested to be used in disseminating information about AS-AQ. It was however observed that community member had difficulty pronouncing AS-AQ.

Respondents who had been treated with AS-AQ themselves or care-givers whose children had previously been treated with AS-AQ perceived AS-AQ to be a good drug. This is because they had observed that it took a long time for them or their children to have another malaria attack following an initial treatment. They attributed this perception to the effectiveness of the drug. The need to make AS-AQ available at the community level for treatment of malaria was emphasised during the FGDs. These themes are illustrated below.

"*The drug is good because ever since I used the drug on the child he hasn't experienced fever again, it is three months now." (A Female participant aged 25-34 years in a rural area)*

"Because of hardship, some people are not able to go to the hospital. I think if we get some in our community drug store it will help a lot" (A male participant aged between 19-24 years in a rural area)

### Awareness of artesunate-amodiaquine (AS-AQ) among health workers and drug sellers

#### Government sector

Heads and staff of all government health facilities including district hospitals, health centres and community clinics were aware of AS-AQ and were already prescribing the drug to their clients. It was observed that, each of the clinics in the study area had a treatment chart which was said to be very helpful in determining the dosage for clients based on their ages and weights.

#### Formal Private sector

Private clinics headed by nurses or doctors and chemical sellers were well informed about the policy change from chloroquine to AS-AQ. They were also aware of the rationale for this policy change and had practical experiences of increasing treatment failure with the continual use of chloroquine. Their information about AS-AQ was through periodic updates by the Pharmacy Council of Ghana and the district health directorates.

#### Traditional sector

Traditional healers were aware of AS-AQ but did not know about the details of the dosage and their side-effect profile. They had however heard that patients who took AS-AQ had headaches. Traditional healers referred patients who complained about any side-effect after taking AS-AQ back to the government health facilities where the drug was obtained. They were willing to refer illnesses that they could not deal with to the clinic for further management.

"Some of my patients bring some (AS-AQ) from the hospital.... What I can say about the drug is that when it came on board some people were complaining of headache and so on but since then I haven't head anything about it again... I don't prescribe AS-AQ but if I realize that the person's illness is above me, then I direct the person to hospital". (A Traditional healer in an urban area)

Both private and public health workers applauded the change in anti-malarial policy from the use of chloroquine to AS-AQ; their main worry was about the reported side-effects: body weakness and headache which they attributed to amodiaquine. Some health workers admitted to administering lower doses of amodiaquine in order to minimise side-effects. Additionally, they advised their clients to eat adequately before taking AS-AQ to prevent general weakness which was thought to be the commonest side-effect.

### Motivational factors to use AS-AQ

**Figure 1:**
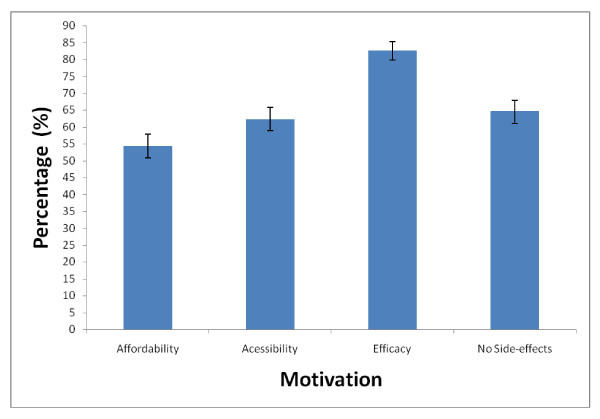
**Factors that motivate household heads and members (N = 807) to use artesunate -amodiaquine in 2006 survey**.

The motivation to use of AS-AQ by household heads and members (N = 807) were assessed in the survey. The perceived efficacy of AS-AQ was the highest (82.7%, 95% CI 79.9 - 85.2) motivating factor for its use, whilst affordability was lowest (54.3%, 95% CI 50.8 - 57.8) (Figure 1).

### Availability and Use of AS-AQ and other antimalarials

The commonest anti-malarial drugs observed in rural chemical shops were *chloroquine (CQ)*; *kinaquine 4-4-2 (KQ) *which is brand of *chloroquine; Sulfadoxine-Pyrimethamine (SP) *in brands such as *Malafan or Fansider*; and *amodiaquine (AQ)*. Chemical sellers identified ' high cost' as a barrier to stocking AS-AQ since their clients found it expensive to buy. They mentioned chloroquine, *Malafan *and *Kinaquine 4-4-2 *as the antimalarials commonly sold within the last three months. The cost of treatment dosage of these drugs varied significantly; USD $0.10 for *CQ *and USD $0.60 for *SP. The cost of brands of artesunate alone found in urban chemical shops ranged between USD 3.00 and USD 3.50*

**Figure 2:**
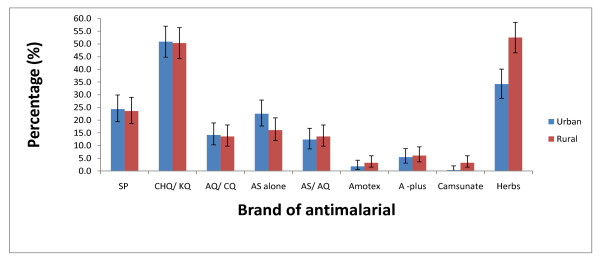
**Drugs used by household heads in urban (N = 275) and rural areas (N = 280) for treating their last episode of malaria in 2006 survey**.

**Figure 3:**
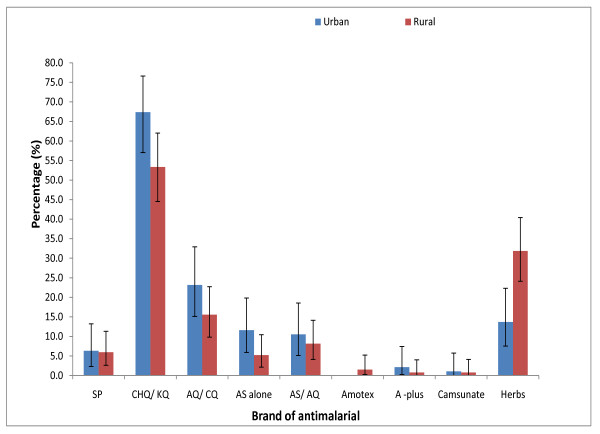
**Drugs used by care-givers in urban (N = 95) and rural (N = 135) areas for treating their children's last malaria episode in 2006 survey**.

Chloroquine/*Kinaquine *were found to be the commonest antimalarial drugs used by household heads (urban = 50.9% (95% CI 44.8 - 57.0), rural = 50.4% (95% CI 44.3 - 56.4) and care-givers (urban = 67.4% (95% CI 57.0 - 76.6), rural = 53.3% (95% CI 44.6 - 62.0 ) (Figures 2 and 3).

The use of AS-AQ was comparatively uncommon in both urban and rural areas; 12.4% (95% CI 8.7 - 16.8) of household heads in the urban and 13.6% (95% CI 9.8 - 18.1) in the rural; for care-givers 10.5% (95% CI 5.2 - 18.5) of the urban and 8.1% (95% CI 4.1 - 14.1) of the rural folks had used the AS-AQ available in health facilities. The use of other brands of AS-AQ combination such as *Amotex, A-plus *and *Camsunate *were uncommon (< 7.0% in all cases). However among these uncommonly used brands, *A-plus *was frequently used among the respondents (household head: urban = 5.5% (95% CI 3.1 - 8.8), rural = 6.1% (95% CI 3.6 - 9.5); care-givers: urban = 2.1% (95% CI 0.2 - 7.4), rural = 0.7% (95% CI 0.0 - 4.1) ).

The use of herbal treatment was most popular in the rural areas as 34.2% (95% CI 28.6 - 40.1) of the urban household heads still use herbs for malaria treatment compared with 52.5% (95% CI 45.8 - 57.8) of rural household heads; for care-givers, 13.7% (95% CI 7.5 - 22.3) of urban compared to 31.9% (95% CI 24.1 - 40.4) of the rural folks still use herbs.

### Challenges in delivering artesunate-amodiaquine (AS-AQ)

AS-AQ is administered on the bases of age and weight and requires the use of birth date records and weighing scales. Poor quality weighing scales were identified by health workers as a challenge at the health facilities in the districts. Another challenge faced by health workers is the act of breaking tablets into smaller bits to get the right proportions for varying weights and ages.

"In fact, all the health facilities have weighing scales only that they are not durable. They are bathroom scales. We keep buying and buying. We need the solar type which is so expensive and only available at some UNICEF programmes" (A health worker in charge of a health policy implementation)

"The problem was the problem of divisibility. As I said earlier on, when I came back from the training, I was a little bit confused on the division aspect of the dosage but now the availability of the chart am okay with that. (A health worker in charge of a health facility)"

#### Side-effect Surveillance records

Both government and private health facilities and drug shops that stocked AS-AQ did not keep any records on known or reported side-effects.

## Discussion

In the 1990's several studies in Ghana and other African countries demonstrated poor knowledge about the causation of malaria leading to gross socio-cultural influence in household malaria treatment behaviour [12,13]. For instance in Ghana, malaria transmission was associated with various misconceptions. Mosquito bites were associated with skin rashes whilst the nuisance made from hammering of the mosquito with sleep disturbances; hardly was the mosquito associated with malaria [14]. The misconceptions about causes of malaria such as standing in the sun, eating oily foods held by the community members in our study area are very similar to those reported about 20 years ago in southern Ghana with similar socio-cultural settings [15]. It is however encouraging to note that majority of community members currently associate malaria transmission with the mosquito. The massive educational campaigns about malaria and its control measures adopted as part of the Roll Back Malaria strategies throughout Ghana may have contributed to this as evident by a significant increase in ITN use in recent years in the study area; from 10% in 2003 to about 60% in 2007, [10]. The majority of care-givers associated high body temperature with malaria. This finding is particularly important and suggests care-givers can identify early symptom of malaria among children and promptly seek care and appropriate therapy with ACTs if available, accessible and affordable. The malaria treatment practice of using left over antimalarials at home identified among respondents could lead to sub-optimal treatment of malaria and resistance to ACTs. Educational campaigns need to be intensified and sustained in malaria control programs.

Awareness of any health intervention greatly influences an individual's health care-seeking behaviour. We recognise that since this study was conducted three to four years ago some of the perceptions and behaviours reported here might have changed over time. Nevertheless, majority of respondents in this study were not aware of AS-AQ as the new anti-malarial drug in place of chloroquine. Awareness in the sampled population conducted in 2006 and the district wide surveys conducted in 2007 was notably different. It is unclear why this differential in awareness was found at these two time points but it may be attributed to differences in knowledge of the respondents in the surveys, a methodology limitation; the 2006 survey respondents targeted heads of households as compared to the 2007 survey where any adult member of the household was allowed to respond on behalf of the household head.

Literacy among community members studied was low; as such majority of participants were not able to mention the name "artesunate-amodiaquine" correctly. This finding is likely to be the same in most rural areas in sub-Saharan Africa. A sustainable multi-prong, population specific approach needs to be adopted especially in areas where literacy is low if a maximum coverage of education about AS-AQ is to be achieved. An acronym for AS-AQ that is easily pronounced and recalled has to be introduced so that it can be used as a household cliché. This method of publicity has been used as educational tools in health programmes such as the "he-ha-ho" communication campaign launched in Ghana to improve home-based care of childhood diseases including malaria [16]. A pre-tested well-packaged message will generate discussions among individuals in the community and propagate information on AS-AQ.

A successful implementation of the change process from monotherapy to ACTs depends on people's perception about the side-effects to a treatment drug. Population perceptions have been known to adversely affect the implementation of health programmes such as polio vaccinations in northern Nigeria [17] and de-worming programme in Ghana [18]. In the year 2005, there was a media outcry about the side-effects of AS-AQ in Ghana. This led to the withdrawal of some brands of AS-AQ from the market [19] and may have affected public confidence, especially in the cities in Ghana. In the study area, the main side-effects reported about AS-AQ by community members include body weakness and dizziness. This is similar to adverse event profile documented in controlled trials of AS-AQ [20]. Effective educational messages about AS-AQ and its side-effects should be maintained to ensure a positive perception about AS-AQ in the community. The perception of health workers about a drug could greatly influence its prescription patterns. Health practitioners generally perceived AS-AQ as a drug with some serious side-effects. Some practitioners either refused to prescribe AS-AQ to their patients or sometimes reduced the dosage of the drug for their clients as a way of preventing or minimising its side-effects. This practice could lead to partial treatment and faster growth of the parasite(s) resistance to ACT's. There is an urgent need to continually monitor branded AS-AQ for their quality due to earlier experience of having to withdraw certain brands of AS-AQ from the Ghana market. The affected manufacturers may find ways of re-introducing their rejected brands into the communities and the reactions earlier experienced could be re-visited.

Availability of drugs and treatment options are crucial for malaria control. One of the policies within the process of change in Ghana was to dispense AS-AQ combination through the government health facilities to ensure accountability and correct usage. For instance, in rural areas of Ghana, the drugs were dispensed in the government rural clinics and not community chemical shops which are the first point of call for malaria treatment in the study areas. This could explain why the drugs were available in the health institutions at the time of the survey and not in community chemical shops which are accessible to community members and may have contributed to the low usage of AS-AQ allowing for the high use of chloroquine and herbal preparations in our rural communities. The availability of AS-AQ and other ACTs in the local chemical shops was found to be persistently low as at January 2008 by USAID | DELIVER Project, Task Order 3, and MSH/SPS Program which assessed antimalarial drug supply chain in Ghana [21]. This pattern of low usage of AS-AQ in the study area are similar to what has been reported in other parts of Africa such as Burundi [22] after AS-AQ implementation as first-line drug for treating malaria.

Home management of malaria with ACTs including AS-AQ has been found to be feasible in Ghana where AS-AQ is now registered to be served over the counter without a prescription [23-25]. Deployment of AS-AQ at the community level through chemical shops and Community Health Planning Service (CHPS) compounds would therefore improve accessibility of the drug at the community level. There is however the need to increase availability of malaria diagnostic tools at the community level. About 37.8% (95% CI 32.9%-42.7%) of all fevers reported in a Mozambique national malaria survey were estimated to be due to malaria [26]. The other febrile illnesses such as respiratory tract infections of bacterial or viral origin could contribute the higher proportion of all fevers. This proportion of fever due to non-malaria diseases is likely to increase over time given the current commitment to malaria programmes that are targeting malaria elimination.

Rapid Diagnostic Tests (RDTs) for malaria using antigen-based dipsticks have the potential to improve diagnosis of malaria and thereby reduce wastage of antimalarial drugs [27]. A combination of RDT plus ACT would improve not only the treatment of malaria but also the clinical management of febrile illness in general; particularly pneumonia which is often misdiagnosed as malaria in children [28] This would also reduce the amount of antimalarial used and thus reduce the drug pressure and selection of drug resistance. Studies are currently being conducted in Kintampo, Ghana to investigate the benefit or otherwise of restricting ACT treatment to diagnosed (RDT or microscopically) malaria.

The high cost of AS-AQ in the private non-government sector was a barrier to access. It is believed that this barrier will be minimized with an increase in National Health Insurance coverage in Ghana which is currently about 38% [29]. Additionally existing and new funding mechanisms such as the Affordable Medicines Facility -malaria (AMFm) hosted by the Global Fund to fight AIDS, Tuberculosis and Malaria seek to make ACTs including AS-AQ affordable (about 0.05 USD) in the private and public sectors [30]. However funding mechanisms are usually time bound and its sustainability are subject to various factors including availability of funds and implementation performance. For instance the AMFm facility is currently set up to run for a period of only 24 months and may be renewed for the countries currently involved or extended to other endemic countries in 2012 [30]. There is the need for endemic country governments and their partners to guarantee the sustainability of affordable ACTS to ensure availability and accessibility in the private sector such as chemical shops located within the communities.

The need to provide accessible alternatives to the group of patients who react to AS-AQ is crucial as this would prevent patients from treating themselves with monotherapies as an alternative treatment to AS-AQ. The Ghana policy currently recognises artemether-lumefantrine as an alternative first-line malaria treatment drug so that those who are unable to cope well with AS-AQ have an alternative. While ensuring the availability of AS-AQ at the community level, an effective pharmacovigilance system needs to be in place to document rare adverse events of AS-AQ. Additionally, an effective monitoring of brands of AS-AQ on the market will be necessary to prevent flooding of the market with unapproved AS-AQ drugs.

## Limitation

Participant understanding of the term 'malaria' could vary among respondents. In this study focus group discussions were first held with community members to assess the local names and descriptions of malaria. These discussions helped to adequately establish local signs and symptoms that describes the biomedical syndrome of malaria. The local names and descriptions of malaria were used to develop quantitative tools. This therefore limited the possibility of participants misunderstanding the term "malaria" for other illnesses. Pictures of common antimalarial drugs were provided to respondents as aids to elicit the type of antimalarial drugs used by community members for their last malaria episode. Additionally, observations of left over antimalarial packages were made. This reduced the possibility of participant response biases arising from the inability to pronounce names of anti-malaria drugs used and time lapse (2 months) between time of last episode of malaria and time of survey. Responses to treatment practices and awareness of AS-AQ may have been influenced by recall biases.

## Conclusion

In conclusion, the knowledge of fever as a symptom of malaria is high among the study population. The awareness of AS-AQ therapy and its side-effect is low in the study area. Community education and sensitization targeting all categories of the population has to be intensified to ensure an efficient implementation process. The useful experiences and lessons learnt by Ghana in this drug policy change process should be an experience that could be utilised by other endemic countries that are in the process of changing their anti-malarial drug policies. We recognise this study was carried out just two years after the policy change. Several experiences may unfold over time and need to be documented carefully so as to help steer the maximum compliance in the use of the AS-AQ and other ACTs in endemic countries.

## Competing interests

The authors declare that they have no competing interests

## Authors' contributions

Conceived and designed the study: KPA, LA, RO, CBP, SOA. Data acquisition: KPA, LA, RO, OB, CZ, AS, SAE. Analyzed the data: KPA, LA, EA, RA, EM. Wrote the manuscript: KPA, LA, SOA. Reviewed manuscript for intellectual content: OB, RO, SS, CBP, DC, SOA. All authors read and approved the final manuscript

## Pre-publication history

The pre-publication history for this paper can be accessed here:

http://www.biomedcentral.com/1471-2458/10/409/prepub
